# Supplementary material for: Empirical methods for controlling false positives and estimating confidence in ChIP-Seq peaks
Source: BMC Bioinformatics. 2008 Dec 5;9:523. doi: 10.1186/1471-2105-9-523 (PMC2628906; doi:10.1186/1471-2105-9-523)
Supplement: Additional File 1 — A variety of html documents from the USeq web site detailing the available applications, their best usage, output file type descriptions, command line menus, etc. [file 1471-2105-9-523-S1.zip › USeqUserGuides/JavaDoc.zip!/JavaDoc/trans/main/MultiSGRPrinter.html]

MultiSGRPrinter


|  |  |  |
| --- | --- | --- |
| **Overview** \| **Package** \| **Class** \| **Use** \| **Tree** \| **Deprecated** \| **Index** \| **Help** | |  |
| **PREV CLASS**   **NEXT CLASS** | **FRAMES**    **NO FRAMES**     **All Classes** |  |
| SUMMARY: NESTED \| FIELD \| CONSTR \| METHOD | DETAIL: FIELD \| CONSTR \| METHOD |  |


---


## trans.main Class MultiSGRPrinter

```
java.lang.Object
  trans.main.MultiSGRPrinter
```

---

``` public class MultiSGRPrinter extends java.lang.Object ```

Prints a multi sgr file (chr position val1, val2, val3, ....)

---

**Constructor Summary**

`MultiSGRPrinter(java.lang.String[] args)`


| **Method Summary** | |
| --- | --- |
| `static boolean` | `checkSizes(float[][] replicasValues)`             Checks to see if the number of values are the same given float[replica#][values]. |
| `static float[][]` | `fetchIntensities(java.io.File[] directories, java.lang.String fileName)`             Fetches a float[] from each of the directories given a fileName. |
| `static void` | `main(java.lang.String[] args)` |
| `static void` | `printDocs()` |
| `void` | `processArgs(java.lang.String[] args)`             This method will process each argument and assign new varibles |

**Methods inherited from class java.lang.Object**

`equals, getClass, hashCode, notify, notifyAll, toString, wait, wait, wait`

**Constructor Detail**

### MultiSGRPrinter

```
public MultiSGRPrinter(java.lang.String[] args)
```


**Method Detail**

### main

```
public static void main(java.lang.String[] args)
```

---


### processArgs

```
public void processArgs(java.lang.String[] args)
```

:   This method will process each argument and assign new varibles

---


### printDocs

```
public static void printDocs()
```

---


### fetchIntensities

```
public static float[][] fetchIntensities(java.io.File[] directories,
                                         java.lang.String fileName)
```

:   Fetches a float[] from each of the directories given a fileName. Returns null
    if a file cannot be read or it is not a serialized float[].

    :   **Returns:**: float[replicas][intensities]

---


### checkSizes

```
public static boolean checkSizes(float[][] replicasValues)
```

:   Checks to see if the number of values are the same given float[replica#][values].


---


|  |  |  |
| --- | --- | --- |
| **Overview** \| **Package** \| **Class** \| **Use** \| **Tree** \| **Deprecated** \| **Index** \| **Help** | |  |
| **PREV CLASS**   **NEXT CLASS** | **FRAMES**    **NO FRAMES**     **All Classes** |  |
| SUMMARY: NESTED \| FIELD \| CONSTR \| METHOD | DETAIL: FIELD \| CONSTR \| METHOD |  |


---
